# Study on the safety and efficacy of Fu's subcutaneous needling for the treatment of lumbar disc herniation: a systematic review and meta analysis of randomized controlled trials

**DOI:** 10.3389/fneur.2025.1509291

**Published:** 2025-04-15

**Authors:** Jiao Liang, Jin Zhang, Jie Zhou, Kun Yang, Qian Xiong

**Affiliations:** ^1^College of Traditional Chinese Medicine, Chongqing Three Gorges Medical College, Chongqing, China; ^2^Department of ENT, Sichuan Provincial Construction Hospital, Chengdu, Sichuan, China

**Keywords:** Fu's subcutaneous needling, lumbar disc herniation, pain management, clinical research, efficacy evaluation

## Abstract

**Purpose:**

Systematic evaluation of the effectiveness and safety of Fu's Subcutaneous Needling (FSN) in the treatment of Lumbar Disc Herniation.

**Methods:**

A systematic search was conducted across four Chinese and four English databases, including China National Knowledge Infrastructure (CNKI), Wanfang, China Science and Technology Journal Database (VIP), China Biology Medicine (CBM), PubMed, Cochrane Library, Embase, and Web of Science, to collect randomized controlled trials (RCTs) on the use of Fu's subcutaneous needling for the treatment of lumbar disc herniation published before September 1, 2024. The search was conducted in both Chinese and English, with no restrictions on ethnicity. After rigorous screening of the literature, Meta-analysis was performed using Stata 18.0 and RevMan 5.2.1 software. This study protocol has been registered with the PROSPERO International Prospective Register of Systematic Reviews, with a registration number CRD42024595890.

**Results:**

A total of 17 studies involving 1,467 patients were included. The Meta-analysis results indicated that Fu's subcutaneous needling for lumbar disc herniation was more effective than the control group, with a statistically significant difference. The overall effective rate was: OR = 2.77, 95% CI (1.90, 4.03), *Z* = 5.31, *P* < 0.00001. The VAS score was: MD = −1.12,95% CI (−1.35,−0.89),*Z* = 9.57,*P* < 0.00001. JOA scores was MD = 4.52, 95% CI (1.83, 7.2), *Z* = 3.29, *P* = 0.001.ODI scores with MD = −6.75, 95% CI (−8.42, −5.08), *Z* = 7.91, *P* < 0.00001. SF-36 with MD = 8.51, 95% CI (3.64, 13.38), *Z* = 3.42, *P* < 0.0006.

**Conclusion:**

FSN has certain advantages and more safety in the treatment of LDH. However, due to the publication bias, the strength of the evidence is insufficient. High-quality, large-sample, multi-center randomized controlled trials are still needed for further research.

**Systematic review registration:**

The protocol for this systematic review was registered on PROSPERO and is available in full on the website (https://www.crd.york.ac.uk/PROSPERO, CRD42024595890).

## Background

Lumbar Disc Herniation (LDH) is a common musculoskeletal condition with a global average incidence rate of 2–3%, affecting more males than females, with significant socioeconomic impact. In China, the average prevalence of LDH is as high as 8–25% (1). The symptoms include low back pain and sciatica, lower extremity weakness, sensory disturbances, or even bowel and genital dysfunction (2, 3). Approximately two-thirds of adults have experienced low back pain. With changes in lifestyle, such as prolonged sitting, obesity, and improper force use, the incidence of LDH is gradually increasing, and the age of onset is advancing, significantly affecting people's quality of life and health. In severe cases, it can lead to the loss of working ability among the working population, causing significant negative impacts on families and the socio-economy (4–6).

Current clinical guidelines recommend non-surgical treatment as the first-line therapy for LDH, with over 80% of LDH patients achieving significant improvement or complete resolution of symptoms through conservative management, which can quickly alleviate patient suffering and promote the recovery process (7, 8). Traditional Chinese medical methods such as warm acupuncture, massage, and spinal manipulation have shown significant improvements in LDH symptoms (9, 10) and are recommended by multiple guidelines (11–14).

Fu's Subcutaneous Needling (FSN) developed by Prof. Fu Zhonghua in 1996, mechanically manipulates fascia through sweeping motions to alleviate neuropathic pain (15). Its analgesic mechanism involves biomechanical forces that reduce fascial tension, reorganize collagen into liquid crystalline arrays, and enhance fascial sliding, thereby breaking the “pain-spasm-ischemia” cycle (16–19). Li et al. found that FSN can enhance the morphological structure and function of mitochondria in tightened muscles, increasing mitochondrial creatine synthase and Complex II levels and boosting the active expression of cytochrome c oxidase (COX-I) protein in muscle tissues (20). Chiu et al. found FSN effectively ameliorates peripheral neuropathic pain by modulating inflammatory responses and endoplasmic reticulum (ER) stress (21).

Compared with traditional acupuncture, FSN exhibits three major characteristic differences: first the target of action focuses on the fascia-nerve interface rather than the meridian acupoints, and it does not elicit the de qi response (22). Second the effective substances rely on cell membrane tension changes induced by mechanical stress rather than pure nerve signal conduction (19). Third the sustained therapeutic effect originates from fascial structural remodeling (15, 17).

Although the earliest literature documenting its use in LDH dates back to 1998, and its efficacy was found to be superior to that of the acupuncture group (23). However, the deadline for inclusion in the literature is August 2022, and the search database is limited, and insufficient analysis of relevant outcome indicators (24, 25). Therefore, it is necessary to expand the search to more databases and conduct a comprehensive systematic review of relevant outcome indicators once again.

## Method

### Search strategy

A comprehensive search was conducted across four Chinese databases (CNKI, WanFang, VIP, and CBM) and four English databases (PubMed, Cochrane Library, EMBASE, and Web of Science).We conducted a comprehensive search for articles on the treatment of “lumbar disc herniation” using “floating needles” in these databases. For the Chinese search terms, we used subject searches with “Floating Acupuncture” and “lumbar disc herniation.” For English searches, we conducted full-text searches using the terms “Floating Acupuncture,” “Floating needle,” “Float needle,” “Float therapy,” “Fu's Subcutaneous Needling,” “Fu's Acupuncture,” and “FSN.” Additionally, we employed “lumbar disc herniation” and “Intervertebral disc displacement” as English search terms for full-text searches. Taking the PubMed search query as an example: “(((((((Floating Acupuncture[Title/Abstract]) OR Floating needle[Title/Abstract]) OR Float needle[Title/Abstract]) OR Float therapy[Title/Abstract]) OR Fu's Subcutaneous Needling[Title/Abstract]) OR Fu's Acupuncture[Title/Abstract]) OR FSN[Title/Abstract]) OR fuzhen[Title/Abstract]) AND lumbar disc herniation[Title/Abstract].” The search covered the publication period from inception to September 1, 2024.

#### Inclusion criteria

(1) The study must be conducted as a randomized controlled trial. The subjects of the study must consist of patients diagnosed with LDH. (2) The intervention measures should entail the experimental group receiving solely FSN, whereas the control group may receive electroacupuncture, acupuncture, or a combination of other traditional Chinese medical therapies. (3) There must be a clearly stated source of diagnostic criteria or imaging detection methods, such as MRI, CT, or X-ray. (4) The outcome indicators should encompass at least one of the following: clinical total effective rate, Visual Analog Scale (VAS) score, Japanese Orthopedic Association (JOA) score, or Oswestry Disability Index (ODI) score.

#### Exclusion criteria

(1) The experimental group does not receive FSN as the intervention measure. (2) The study subjects are LDH patients who have already undergone surgical treatment. (3) The articles in question are case studies, popular science articles, conference papers, academic theses, mechanism exploration articles, withdrawal statements, or review articles, which do not meet the requirements for original research studies.

### Data collection

Two researchers, JL and JZ, independently screened the literature in two stages. First, they examined the titles and abstracts, and then they reviewed the full text. During this process, they eliminated articles that were clearly irrelevant, including letters, comments, reviews, animal studies, single case reports, observational studies, and meta-analyses. In the event of any disagreements arising during the screening process, a third reviewer, KY, was consulted to resolve the issue.

Basic data extraction encompassed the following elements: (1) The first author's name, publication year, and diagnostic criteria. (2) Research characteristics, including sample size, gender ratio, intervention method, follow-up duration, and whether the FSN group underwent reperfusion or not. (3) Outcome indicators, along with the total number of treatment sessions and the frequency of each session. (4) Factors that impact the quality of the literature.

### Quality assessment

The quality of the included literature was assessed based on the methodological and quality criteria outlined in the Cochrane Handbook for Systematic Reviews (1, 26), with a focus on the following seven aspects: randomization method, allocation concealment, blinding, outcome assessment bias, completeness of outcome reporting, selective outcome reporting, and other potential biases. Each aspect was rated as “low risk,” “high risk,” or “unclear risk,” and a risk of bias graph was subsequently generated. This evaluation process was independently conducted and cross-checked by two researchers, JL and JZ. In the event of any disagreements, a third researcher, KY, was consulted to jointly resolve the issues.

### Statistical analysis

The results, which included the efficacy rate, adverse reactions, VAS score, ODI score, JOA score, and SF-36 score, were subjected to meta-analysis using RevMan 5.2.1 software. This process led to the generation of forest plots for these indicators. Heterogeneity was assessed using the *I*^2^ statistic, with values exceeding 50% indicating substantial heterogeneity. For data exhibiting *I*^2^ > 50%, a random effects model was employed for analysis; otherwise, a fixed effects model was used. The forest plots illustrate the calculated outcome measures along with their corresponding 95% confidence intervals (CI). Statistical significance was determined using a *P*-value threshold of < 0.05. Subgroup analysis was conducted based on different treatment methods in the control group, whether FSN has reperfusion design, and different criteria for evaluating effectiveness. Funnel plots were utilized to evaluate publication bias, and sensitivity analysis was conducted using Stata 18.0 software through a sequential exclusion approach.

## Results

### Search results

**Figure 1:**
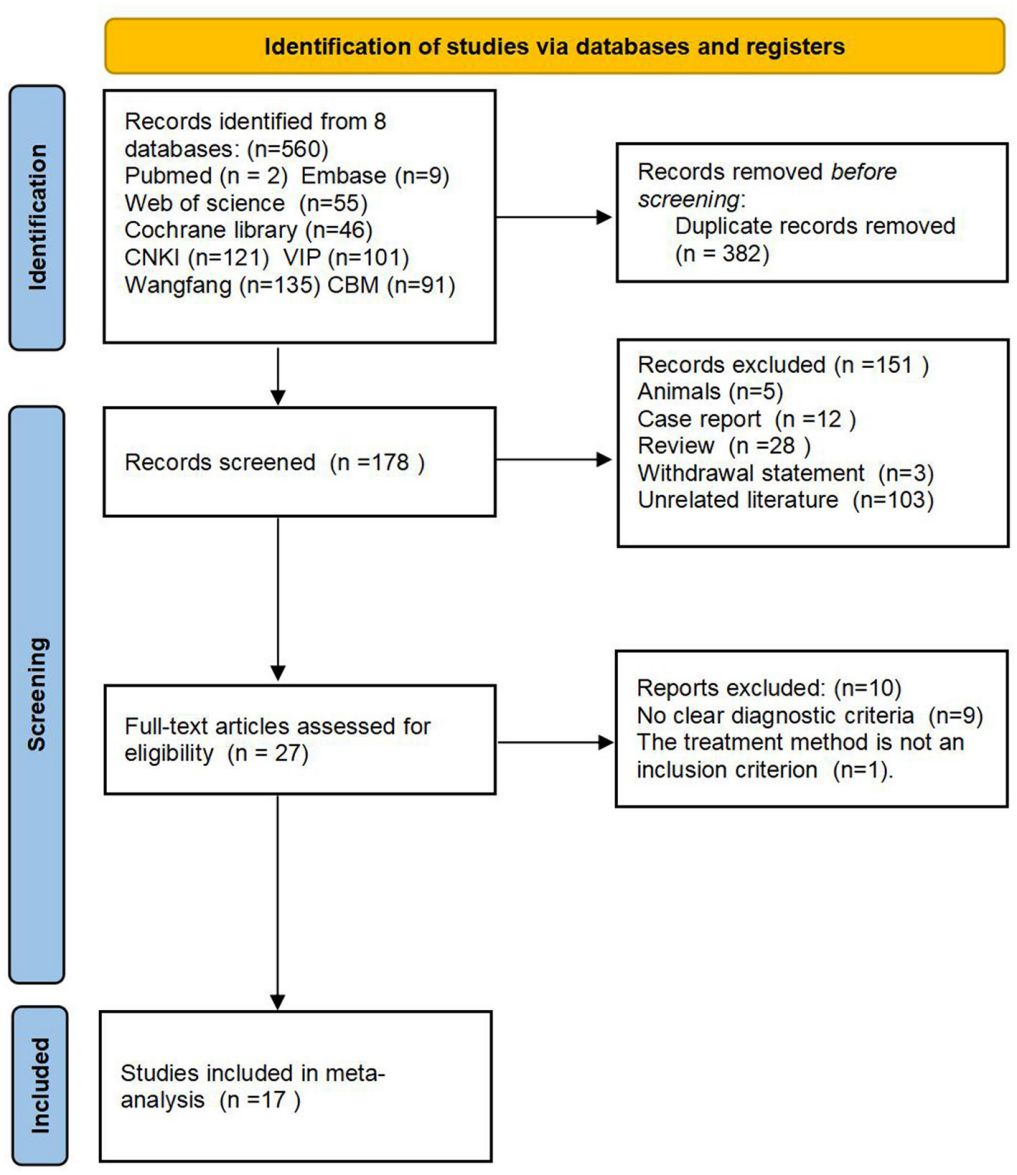
Flow diagram of literature screening and selection outcomes.

An initial search of databases yielded 560 relevant studies. Using EndNote X7, we successfully eliminated 382 duplicate studies. Upon a thorough review of the titles and abstracts of the remaining 178 articles, we excluded 103 unrelated pieces of literature, 28 reviews, 3 withdrawal statements, 5 animal experiments, and 12 case reports. Subsequently, full-text reviews were conducted on the remaining 27 articles. However, 9 of these lacked a clear diagnostic criterion and 1 had an incompatible intervention method in the control group, resulting in their exclusion. Ultimately, 17 clinical studies met the criteria and were deemed suitable for inclusion in the meta-analysis. A detailed flowchart depicting this process is presented in Figure 1.

### Characteristics of the included studies

Based on inclusion and exclusion criteria, 17 articles were selected, spanning from 2001 to 2024. These studies included 1,467 cases, with 736 in the FSN group and 731 in the control group. Among these articles, 12 (27–38) employed the “Diagnostic and Therapeutic Criteria for Traditional Chinese Medicine Diseases and Syndromes” (ZY/T001.9-94) (published in 1994, 2012, and 2017) as their diagnostic benchmark (39). One article (29) referenced “Lumbar Disc Herniation” authored by Hu (40), 1 cited (31) “Orthopedics of Traditional Chinese Medicine” edited by Wang (41), 1 (42) used “Practical Orthopedics” edited by Xu (43), and 1 employs the diagnostic criteria based on “Surgery” edited by Chen (44) and Guiding Principles for “Clinical Research of New Chinese Medicinal Products” edited by Zheng (45) as their standards. Additionally, 12 articles use imaging tests (X-ray, CT, MRI) (23, 27, 28, 30–32, 35–38, 46, 47) as diagnostic criteria.

In interventions, all articles' experimental groups received FSN therapy, while control groups underwent acupuncture (23, 27, 28, 30, 32, 34–38, 42, 46–49) or electroacupuncture (29, 31). Three articles (23, 38, 47) reported follow-up durations, encompassing 12 outcome indicators. The total effective rate was the most commonly reported outcome, appearing in 16 articles (23, 27–38, 42, 46, 47, 49). VAS score in four articles (34, 38, 47, 49), JOA score in 2 articles (34, 38), ODI score in three articles (34, 46, 47), adverse reactions in four articles (37, 38, 46, 47). One article (46) involved hematological indicators, and two articles (38, 47) incorporated quality of life scales (SF-36). The fundamental characteristics of the literature are outlined in Table 1.

**Table 1:** Basic characteristics of included literature.

**Author Year**	**Diagnostic criteria**	**Sample size**		**Male/female**		**Intervention**	**Follow-up time**	**Outcomes**	**Treatment numbers**		**Treatment frequency**		**Reperfusion**

		**FSN**	**C**	**FSN**	**C**				**FSN**	**C**	**FSN**	**C**	
Li 2001	CT	46	50	29/17	28/22	Acupuncture	1 year	(1)	15	30	qod	qd	NO
Xu 2006	CT/MRI,	51	51	64/38		Acupuncture	NA	(1)	10	10	qod	qd	NO
Zhang 2011	CT/MRI,	40	40	24/16	22/18	Acupuncture	NA	(1)	5 or10	10 or 20	qod	qd	NO
Chen 2011	CT/MRI,	50	50	35/15	24/26	Acupuncture	NA	(1)	10	10	qd	qd	NO
Bao 2012		20	28	12/8	16/12	Acupuncture	NA	(1)	10	20	qod	qd	NO
Huang 2015	MRI/X-ray,	51	50	27/24	26/24	Acupuncture	NA	(1)	NA	10 or 20	NA	qd	Yes
Yang 2015	CT/MRI,	90	90	38/52	NA	Acupuncture	NA	(1)	6	10	NA	qd	Yes
Qin 2016	CT,	40	40	21/19	22/18	Electroacupuncture	NA	(1) (2)	10	10	4 days, qd, then qod	qd	Yes
Sun 2019	MRI/CT,	38	38	22/16	23/15	Acupuncture	NA	(1)	6	20	Every 3 days	qd	NO
Li-Y 2020		62	46	35/27	26/20	Acupuncture	NA	(1)	3	10	qod	qd	NO
Yang 2020		40	40	16/24	13/27	Acupuncture	NA	(1) (5) (6) (7)	12	18	3 days, qd, then qod	qd	Yes
Li-W2020		32	32	13/19	11/21	Electroacupuncture	NA	(1)	12	12	qd	qd	Yes
Chen 2022		30	30	17/13	16/14	Acupuncture	NA	(1) (3) (4) (5)	9	18	qod	qd	NO
Li 2022	MRI/CT/X-ray,	40	40	22/18	24/16	Acupuncture	NA	(1) (7) (8)	9	14	3 days, qd, then rest for 2 days	qd	NO
Chen 2023	CT/MRI,	41	41	27/14	25/16	Acupuncture	NA	(1) (7) (8) (9) (10) (11)	10	20	qod	qd	Yes
Sun 2024	CT/MRI,	30	30	17/13	16/14	Acupuncture	6 months	(1) (5) (6) (8) (12)	10	12	3 days, qd,and then qod	qd	NO
Yuan 2024	CT/MRI	35	35	35/0	35/0	Acupuncture	2 weeks	(5) (7) (8) (12)	6	10	tiw	qd	Yes

Diagnostic criteria: “Diagnostic and therapeutic standards for traditional Chinese medicine diseases and syndromes” (ZY/T001.9-94); “Lumbar disc herniation;” “Traditional Chinese medicine orthopedics;” “Practical orthopedics;” “Surgery;” “Guiding principles for clinical research of new traditional Chinese medicine drugs.”

Treatment Frequency: qd, once a day; qod, once every two days; tiw, three times a week; NA, Not Applicable.

Efficacy indicators: (1) Total effective rate; (2) Short form McGill Pain Questionnaire (SF-MPQ); (3) Pain Self Rating Scale; (4) Pain Rating Index (PRI); (5) Visual Analogue Scale (VAS); (6) Japanese Orthopedic Association Evaluation of Treatment Score (JOA); (7) Oswestry Disability Index score (ODI); (8) Adverse reactions; (9) Traditional Chinese Medicine Syndrome score; (10) NRS pain rating scale; (11) Serum inflammatory factors; (12) Quality of life scale (SF-36).

### Quality assessment

**Figure 2:**
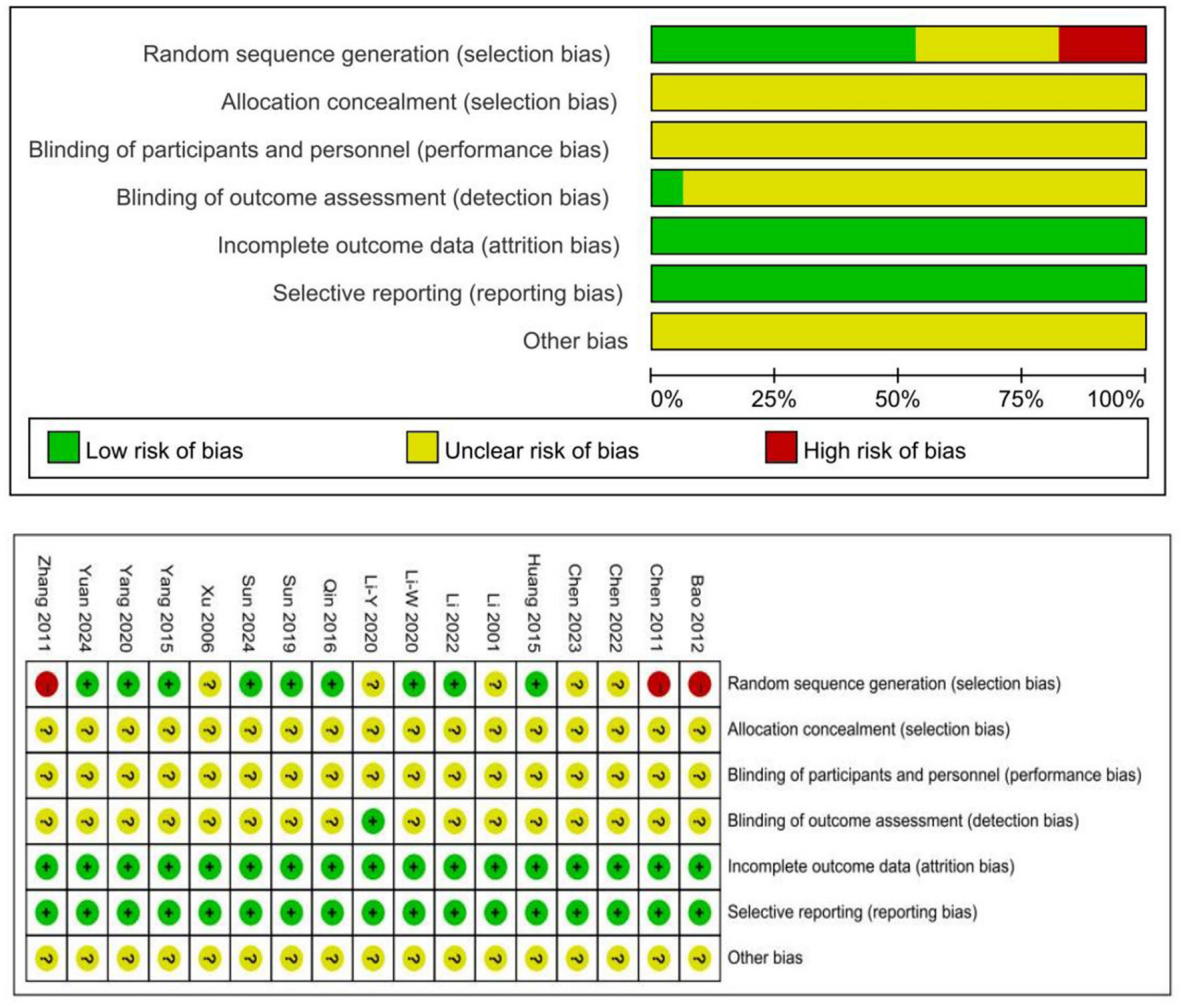
The figure represents the risk of bias assessment for the studies.

Seven articles (28, 32, 34, 35, 38, 47) employed random number generation. Three articles (27, 36, 48) grouped patients according to the sequence of their visits. One article (31) used coin tossing, another (29) utilized dice rolling, and two articles (30, 46) simply mentioned random allocation without elaborating on the specific method used. Three articles (23, 42, 49) did not explain the random methods used. One article (42) adopted a single-blind method for grouping. Three articles (23, 38, 47) provided follow-up results, and four articles (37, 38, 46, 47) reported on adverse reactions. Notably, no studies experienced dropout cases, and all outcomes were thoroughly reported. The outcomes of the methodological evaluation are presented in Figure 2.

### Total effective rate

Among the 17 articles, all except one (47) reported the total effective rate, using various evaluation criteria. Specifically, two articles (27, 48) adopted the “Scoring System for Therapeutic Effects of Lumbar Disorders” formulated by the Japanese Orthopedic Association in 1984, 3 articles (37, 38, 42) utilized the “Guiding Principles for Clinical Research of New Traditional Chinese Medicine Preparations” as the standard, and the remaining articles employed the ZY/T001.9-94 as the evaluation criterion. The heterogeneity test (*P* = 0.70, *I*^2^ = 0%) indicated good homogeneity across studies, a fixed-effects model was used. The difference was statistically significant [OR = 2.77, 95% (1.90, 4.03), *Z* = 5.31, *P* < 0.00001], indicating that the total effective rate in the FSN group was significantly higher than the control group.

**Figure 3:**
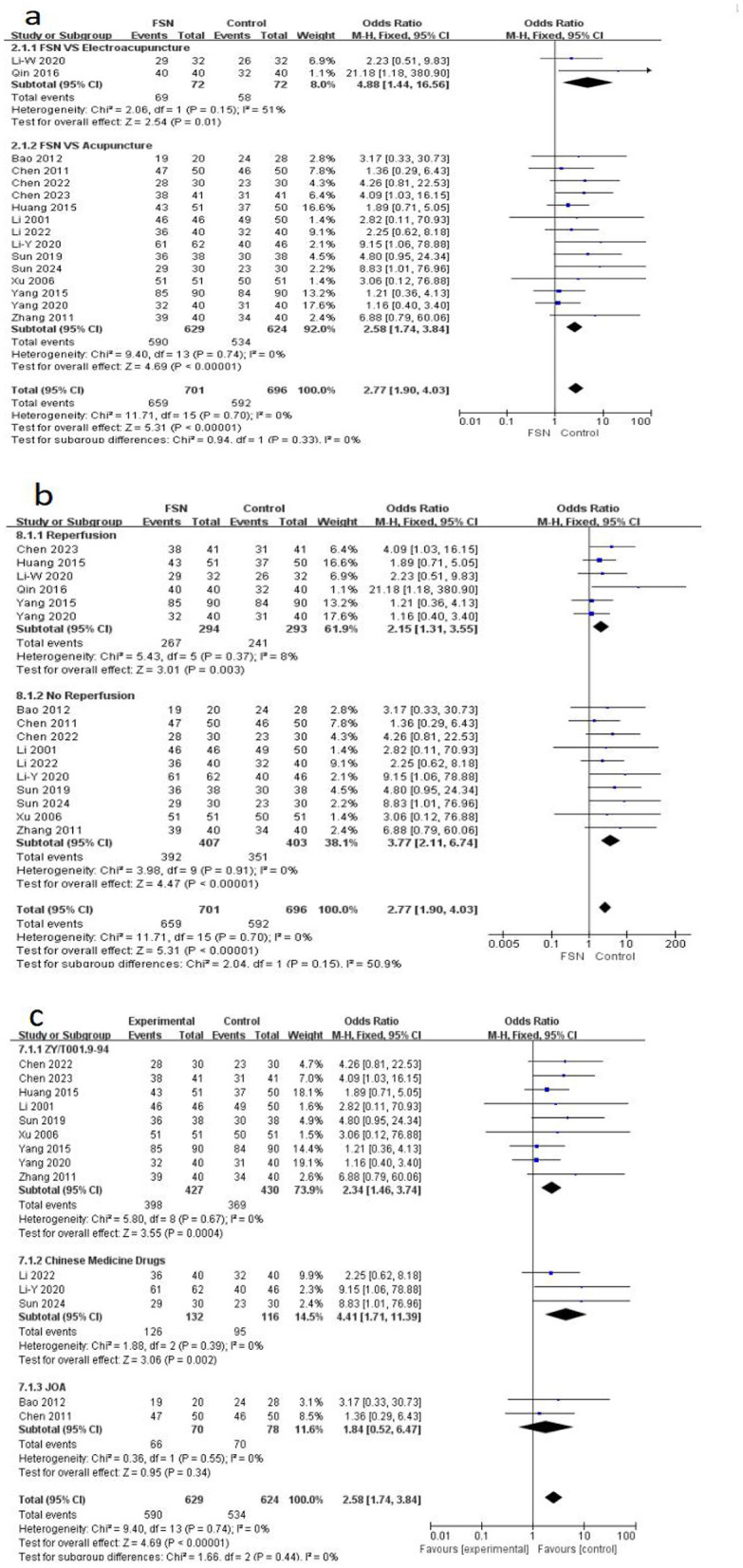
The figure represents a forest plot of subgroup analyses against Total effective rate **(a)**. Forest plots on different intervention type **(b)**. Forest plots on whether the FSN group received re-perfusion. Forest plots on different efficacy evaluation criteria **(c)**.

Subgroup analysis was conducted based on the intervention type of the control group, whether the FSN group received reperfusion, and different efficacy evaluation criteria. The different interventions in the control group are shown in Figure 3a. Acupuncture 14 vs. Electroacupuncture 2. Heterogeneity tests showed good homogeneity among the included studies (*P* = 0.74, *I*^2^ = 0%). Fixed effects models were used for analysis, and the results showed that the total effective rate of FSN treatment was higher than that of the acupuncture control group (*P* < 0.00001). The electroacupuncture group Qin 2016 (31) may be the source of heterogeneity. The heterogeneity tests for the reperfusion (7 studies) vs. non-reperfusion (10 studies) subgroups regardless of whether reperfusion was performed, the FSN group demonstrated generally superior efficacy compared to the control group (*P* < 0.00001), see Figure 3b. The heterogeneity tests for the various evaluation criteria reveal that, despite the inconsistency in the efficacy evaluation indicators employed, good homogeneity is still observed in the subgroup analysis. Furthermore, there is a high degree of consistency between the “Guiding Principles for Clinical Research of New Traditional Chinese Medicine Preparations” and ZY/T001.9-94, as illustrated in Figure 3c.

### VAS scores

**Figure 4:**
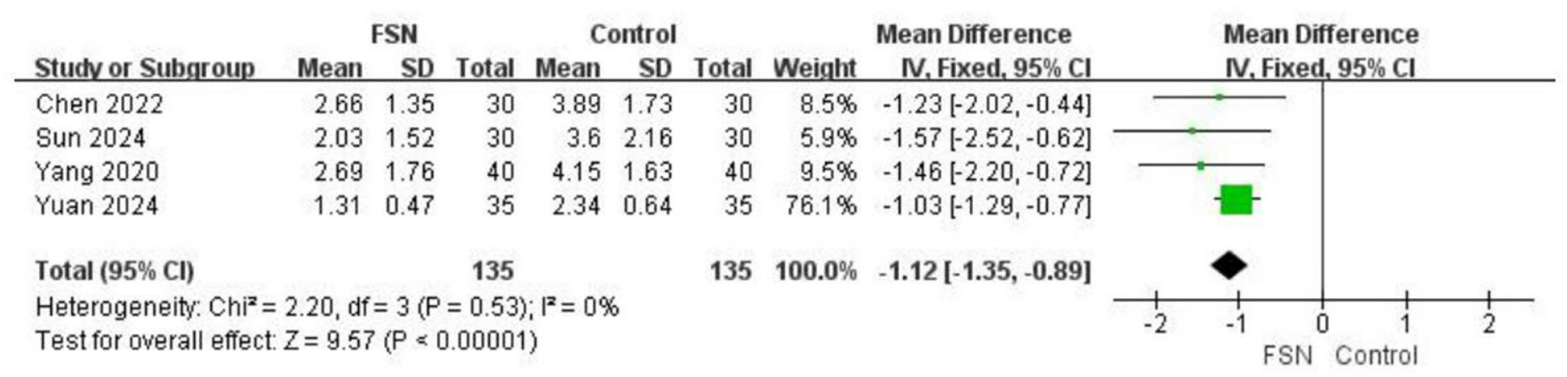
The figure represents a forest plot of VAS scores.

Four studies (34, 38, 47, 49) reported on VAS scores, involving a total sample size of 270 patients, with 135 in the experimental group and 135 in the control group. Heterogeneity testing shows (*P* < 0.00001, *I*^2^ = 0%) good homogeneity among the studies. A fixed-effects model was used for analysis. Floating Acupuncture demonstrates a significant advantage in reducing the VAS scores of LDH patients [MD = −1.12, 95% (−1.35, −0.89), *P* < 0.00001], See Figure 4 for details.

### JOA score

**Figure 5:**
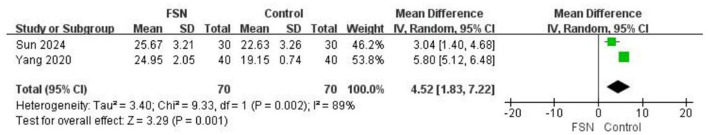
The figure represents a forest plot of JOA scores.

Two studies (34, 38) on JOA scores of 140 LDH patients (FSN people 70), Heterogeneity testing shows (*P* = 0.002, *I*^2^ = 89%) high homogeneity among the studies. A fandom-effects model was used for analysis. FSN group can improves JOA scores of LDH patients [MD = 4.52, 95% (1.83, 7.2), *P* = 0.001], See Figure 5 for details. The heterogeneity between the two studies may be attributed to the different disease durations of the patients included. In Sun's (38) study, all patients were in the acute phase of LDH, whereas in Yang's (34) study, the patients were not in the acute phase.

### ODI score

**Figure 6:**
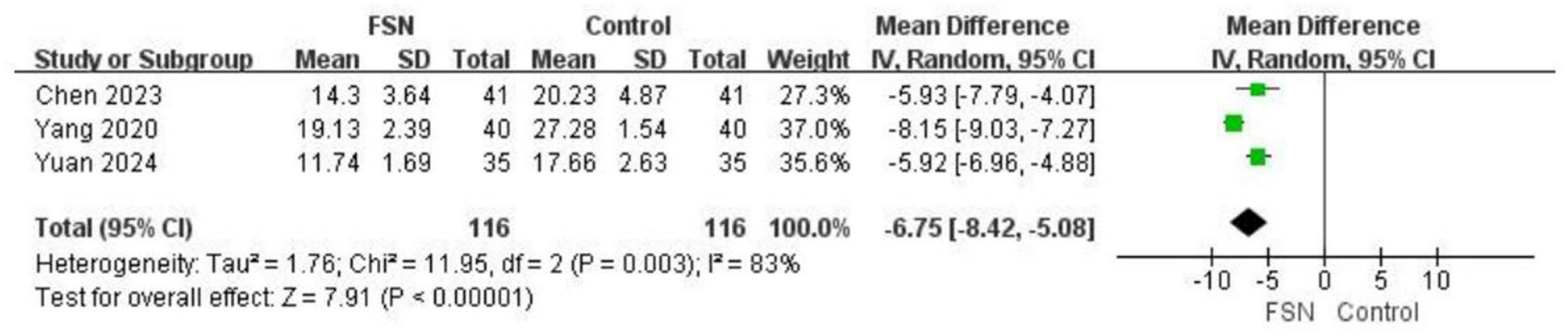
The figure represents a forest plot of ODI scores.

Three studies (34, 46, 47) reported ODI scores of 332 LDH patients (FSN people 116). High heterogeneity (*P* = 0.003, *I*^2^ = 83%) led to random-effects model use. Sensitivity analysis showed Yang 2020 (34) significantly impacted results. FSN group can decrease ODI scores of LDH patients [MD = −6.75, 95% (−8.42, −5.08), *P* < 0.00001]. show in Figure 6.

### SF-36 score

**Figure 7:**
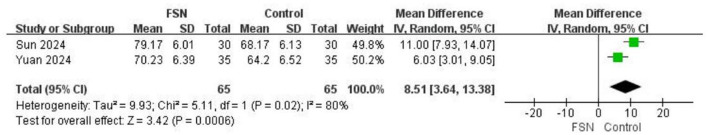
The figure represents a forest plot of SF-36 scores.

Two studies (38, 47) reported SF-36 scores of 130 LDH (FSN people 65). High heterogeneity (*P* = 0.02, *I*^2^ = 80%) led to random-effects analysis. The heterogeneity between the two studies may be attributed to differences in the study populations and variations in the total number of treatment sessions. Using FSN therapy can significantly improves patients' quality of life [MD = 8.51, 95% (3.64, 13.38), *P* < 0.0006], show in Figure 7.

### Adverse reactions

Four articles (37, 38, 46, 47) mention adverse reactions. Li (37) observed 1 ecchymosis, 1 bleeding, 0 hematoma in FSN vs. 2 bleeding, 1 hematoma in control. Chen (46) found fewer adverse reactions in FSN (1 bleeding, 1 needle retention, 1 infection) than control (2 bleeding, 1 needle retention, 2 infection). Sun (38) and Yuan (47) both reported no significant adverse reactions. Overall, the FSN group had fewer adverse reactions than the control the FSN group had fewer adverse reactions.

### Publication bias

**Figure 8:**
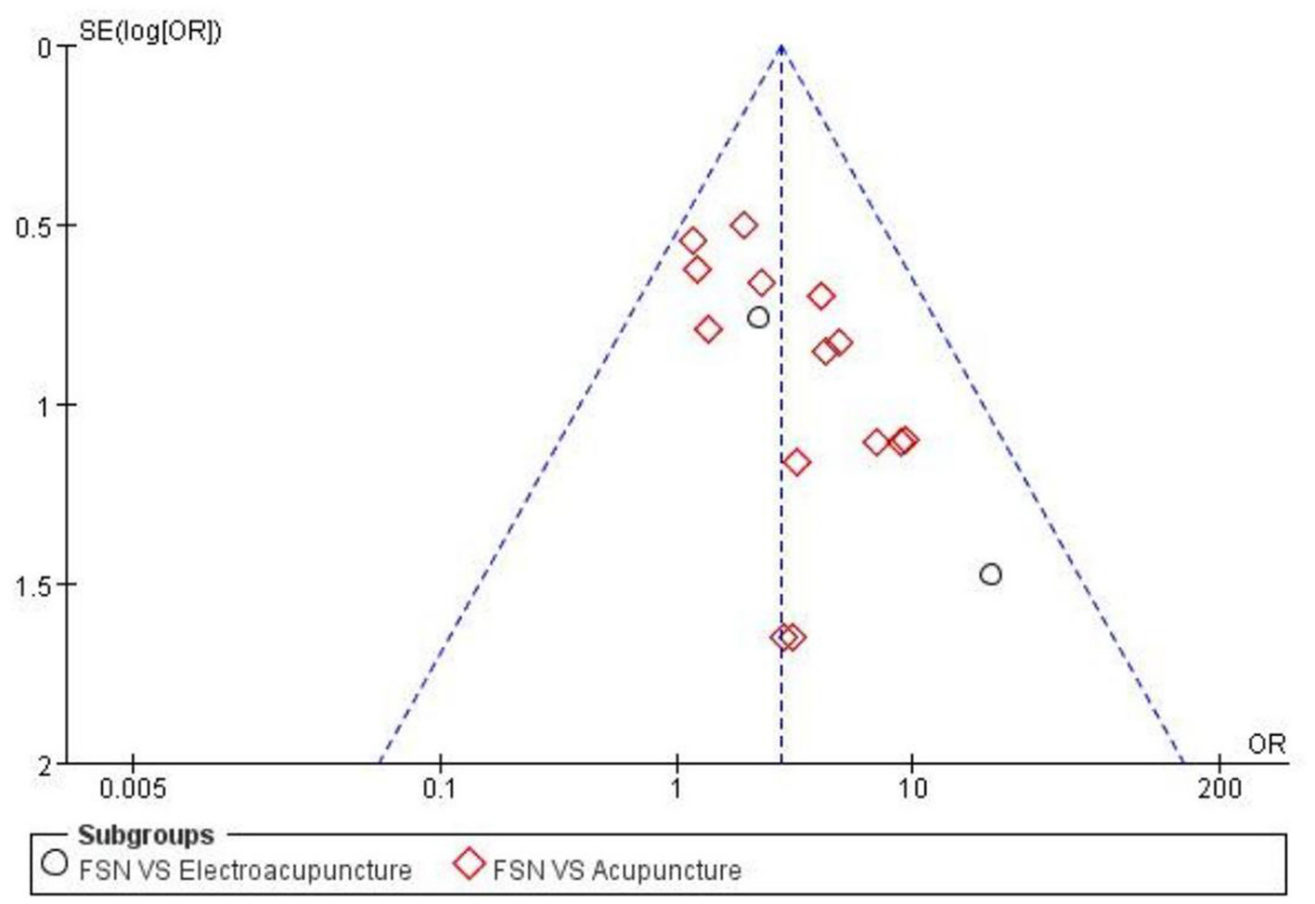
The figure represents the funnel plot.

An inverted funnel plot assessed publication bias among 16 articles on overall effective rate (Figure 8). The funnel plot suggested publication bias, likely due to low article quality and small sample sizes. Egger's test [*P* = 0.004, 95% (0.66, 2.82)] confirmed significant bias.

### Sensitivity analysis

**Figure 9:**
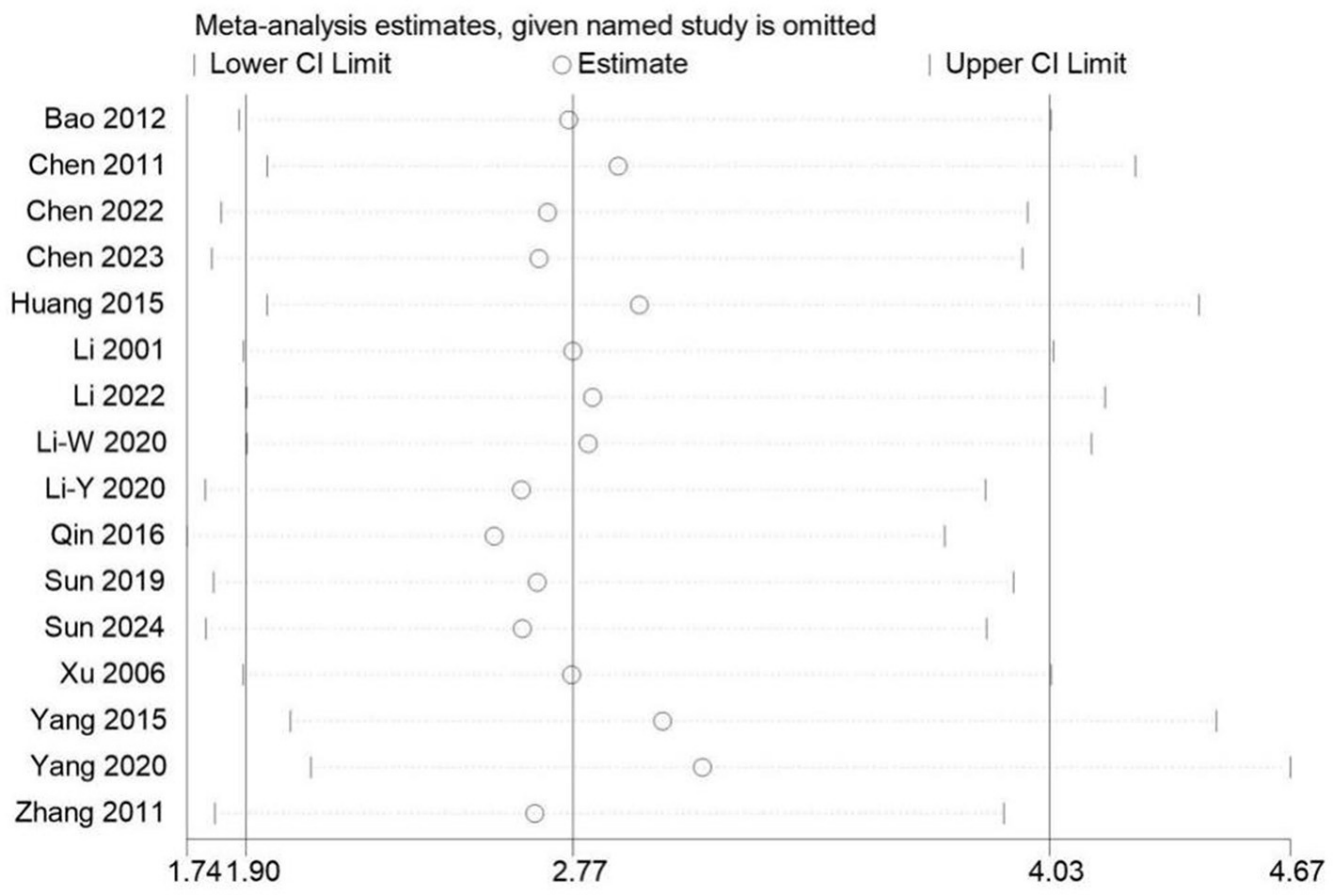
The figure represents the sensitivity analysis.

A sensitivity analysis was conducted on 16 studies reporting the overall effectiveness rate (Figure 9). The total effect size OR = 2.49 exhibited some bias compared to 2.77, however, the 95% CI for OR fell within the range of (1.68, 3.70). There were no studies with particularly significant heterogeneity, indicating that the results were relatively stable.

## Discussion

This meta-analysis of 17 studies (*n* = 1,467) demonstrates FSN significantly outperforms control interventions in LDH management. FSN showed superior overall effectiveness (OR = 2.77, *P* < 0.00001), with clinically meaningful pain reduction (VAS = −1.12, *P* < 0.00001). Functional improvements were evidenced by JOA (MD = 4.52, *P* = 0.001) and ODI scores (MD = −6.75, *P* < 0.00001), while quality-of-life enhancement reached SF-36 (MD = 8.51, *P* < 0.0006). These findings position FSN as a viable non-pharmacological adjunct for LDH, warranting integration into clinical protocols while emphasizing need for long-term follow-up studies.

Fu's Needling Therapy, proposed by Professor Fu Zhonghua (50) in 1996, is a special acupuncture method that uses a specially designed needle to perform sweeping and dispersing stimulation in the subcutaneous fascial layer. It is particularly effective in treating muscle-related pain disorders, characterized by mild pain, rapid onset of efficacy, and a small number of needle insertion points (50–54).

Professor Fu believes that the sweeping and dispersing motion of the needle in the superficial fascia can pull the fascia, relieve compression of horizontal collateral meridians, and establish low-resistance pathways. During treatment designing reperfusion activities for affected muscles can enhanced therapeutic efficacy, promote qi and blood circulation faster, and achieve the goal of treating the disease rapidly (22, 29, 46). Reperfusion therapy is a hot topic in research on Fu's Needling Therapy (55). Professor Fu suggests that appropriate reperfusion of the affected muscle can more effectively promote the qi and blood circulation in the muscle and improve therapeutic efficacy. In subgroup analysis based on whether reperfusion was performed, the Fu's Needling Therapy group showed better therapeutic efficacy than the control group regardless of reperfusion. An independent-sample *t*-test was conducted using Stata software to analyze whether reperfusion therapy was adopted in the literature on Fu's Needling Therapy. The results showed that *t* = −4.2523 and *P* = 0.0000, indicating a statistically significant difference. This suggests that Fu's Needling Therapy combined with reperfusion therapy yields better therapeutic efficacy.

After analyzing the number of treatment sessions in the extracted literature, two articles (28, 36) with unclear information on the number of treatments were excluded. Stata 18.0 was used to conduct an independent-sample *t*-test on the remaining 15 articles (23, 27, 29–32, 34, 35, 37, 38, 42, 46–49). The results showed that *t* = −22.034 and *P* = 0.0000, indicating a statistically significant difference. The total number of treatment sessions for LDH using FSN was significantly fewer than that of the acupuncture group, demonstrating that Fu's Needling Therapy is characterized by a reduced number of treatment sessions. Unfortunately, in the available literature, the treatment sites are typically referred to as tender points, myofascial trigger points, or affected muscles, without providing sufficient information for analysis.

An analysis of the treatment frequency in the extracted literature revealed that the control groups all received treatment once a day. Two articles (28, 35) did not specify the treatment frequency in their text. Seven articles (23, 30, 36, 42, 46, 48, 49) reported a treatment frequency of once every 2 days. Six articles (27, 29, 31, 34, 38, 47) reported a treatment frequency of once a day, but among them, Qin (31) indicated a switch to once every 2 days after 4 days, Yang (34) stated a switch to once every 3 days after 3 days, Sun (38) mentioned a switch to once every 2 days after 3 days, Sun (32) indicated a treatment frequency of once every three days, and Yuan (47) recorded a treatment frequency of three times a week. It can be seen that there are differences in the clinical treatment frequency of FSN. Qin (31), Yang (34), and Sun (38) believed that daily treatment could be given during the acute phase to promptly relieve pain, followed by interval treatment once the pain stabilized. However, there is still controversy regarding the specific interval time, which still requires further study.

Previous research has demonstrated FSN therapy's effectiveness in alleviating various pain conditions (52, 53). In this study, four articles using the VAS score to assess pain in LDH patients found that the VAS scores of the FSN group were better than those of the control group. However, due to differences in the indicators used by researchers to assess pain, such as the SF-MPQ scale used by Qin (31), the hospital pain self-assessment scale used by Chen (49), and the NRS scale used by Chen (46), the application of too many similar scales prevents the merging of similar items. This limitation on the number of studies may result in an objective meta-analysis outcome being unattainable.

The criteria for evaluating the efficacy of FSN therapy in LDH patients also exhibit diversity. The most commonly used standard is the ZY/T001.9-94 while some researchers also adopt the “Guiding Principles for Clinical Research of New Chinese Medicines.” Although some studies have evaluated JOA, ODI, and SF-36 to further supplement the therapeutic effect, the current meta-analysis results show heterogeneity. Due to the limited number of studies included in such data, subgroup analysis and meta-regression could not be performed. A random-effects model was used for analysis, and sensitivity analysis was conducted to exclude the influence of outliers. Analysis of the relevant literature suggests that heterogeneity may stem from clinical factors such as patients' initial disease status, treatment frequency, total number of treatments, and population characteristics. It is recommended that future studies increase sample size and incorporate stratified grouping of these factors in trial designs to clarify the stability of FSN efficacy and its applicable population characteristics.

As an invasive procedure, the safety profile of FSN warrants particular clinical attention. However, only four out of 17 included studies (23.5%) reported adverse events (AEs), with a total population of 146 patients in both FSN and control groups. The AE analysis revealed the following patterns: In FSN group (*n* = 146): total AEs: 5 events (3.4% incidence), bleeding: 2 cases (1.4%), Needle retention: 1 case (0.7%), ecchymosis: 1 case (0.7%), infection: 1 case (0.7%), hematoma: 0 cases. In control group (*n* = 146): total AEs: 8 events (5.5% incidence), bleeding: 3 cases (2.1%), hematoma: 1 case (0.7%), needle retention: 1 case (0.7%), infection: 2 cases (1.4%). The FSN group showed a numerically lower AE incidence (3.4% vs. 5.5%), though the clinical significance of this difference remains uncertain due to limited event numbers. Future research should systematically document adverse events, including severity, and incidence rates.

With the deepening of research, clinical researchers tend to use more indicators to comprehensively assess LDH patients treated with Fu's Needling Therapy, such as the Traditional Chinese Medicine Syndrome Score (46), serum inflammatory factors (46), SF-36 (38, 47), and ODI (34, 38, 46) scales. To provide more and higher-quality clinical studies, it is necessary for relevant associations or guideline proposers to standardize and guide the corresponding evaluation indicators. It is necessary for personnel of the Floating Acupuncture Association to provide clear instructions on the selection of needle insertion points, rather than relying solely on palpation as a subjective assessment. Furthermore, there needs to be more standardization regarding the frequency and number of Floating Acupuncture treatments in order to facilitate its promotion.

## Conclusion

FSN therapy demonstrates significant superiority over the control group in terms of treatment effectiveness, VAS scores, ODI scores, SF-36 score and JOA scores. However, the retrieved literature is limited with low quality evaluations, exist publication bias, and small sample sizes. Additionally, due to the invasive nature of the intervention, blinding of subjects is not feasible. Descriptions of dropout rates during follow-up are also insufficient. Therefore, future FSN trials should incorporate more diverse populations to enhance generalizability, use standardized outcome measures to facilitate comparison across studies, and implement longer follow-up periods to assess the sustainability of interventions. Additionally, we recommend the use of adaptive trial designs to allow for modifications based on interim results, which could improve the efficiency and relevance of FSN research.

## Data Availability

**T**he original contributions presented in the study are included in the article/Supplementary material, further inquiries can be directed to the corresponding author.
