# Supplementary material for: Study on the safety and efficacy of Fu's subcutaneous needling for the treatment of lumbar disc herniation: a systematic review and meta analysis of randomized controlled trials
Source: Front Neurol. 2025 Apr 15;16:1509291. doi: 10.3389/fneur.2025.1509291 (PMC12037388; doi:10.3389/fneur.2025.1509291)

Supplementary materials

[Supplement 1.The risk of bias assessment for the studies 1](#_Toc182689041)

[Supplement 2.Sensitivity analysis of ODI 3](#_Toc182689042)

[Supplement 3.Egger's test 4](#_Toc182689045)

#

# Supplement 1.The risk of bias assessment for the studies

| **NO.** | **First Author** | **Random sequence generation (selection bias)** | **Allocation concealment (selection bias)** | **Blinding of participants and personnel (performance bias)** | **Blinding of outcome assessment (detection bias)** | **incomplete outcome data (attrition bias)** | **Selective reporting (reporting bias)** | **Other bias** |
| --- | --- | --- | --- | --- | --- | --- | --- | --- |
| 1 | Li 2001 | unknow | unclear | unclear | unclear | no | no | unclear |
| 2 | Xu 2006 | Only mentioned random | unclear | unclear | unclear | no | no | unclear |
| 3 | Zhang 2011 | Order of Visit | unclear | unclear | unclear | no | no | unclear |
| 4 | Chen 2011 | Order of Visit | unclear | unclear | unclear | no | no | unclear |
| 5 | Bao 2012 | Order of Visit | unclear | unclear | unclear | no | no | unclear |
| 6 | Huang 2015 | random number generation | unclear | unclear | unclear | no | no | unclear |
| 7 | Yang 2015 | random number generation | unclear | unclear | unclear | no | no | unclear |
| 8 | Qin 2016 | coin tossing | unclear | unclear | unclear | no | no | unclear |
| 9 | Sun 2019 | random number generation | unclear | unclear | unclear | no | no | unclear |
| 10 | Li Y 2020 | unknow | unclear | unclear | Only mentioned single blind | no | no | unclear |
| 11 | Yang 2020 | random number generation | unclear | unclear | unclear | no | no | unclear |
| 12 | Li W Y2020 | dice rolling | unclear | unclear | unclear | no | no | unclear |
| 13 | Chen 2022 | unknow | unclear | unclear | unclear | no | no | unclear |
| 14 | Li 2022 | random number generation | unclear | unclear | unclear | no | no | unclear |
| 15 | Chen 2023 | Only mentioned random | unclear | unclear | unclear | no | no | unclear |
| 16 | Sun 2024 | random number generation | unclear | unclear | unclear | no | no | unclear |
| 17 | Yuan 2024 | random number generation | unclear | unclear | unclear | no | no | unclear |

**Supplement 2.Sensitivity analysis of ODI**

**Supplement 3.Egger's test**


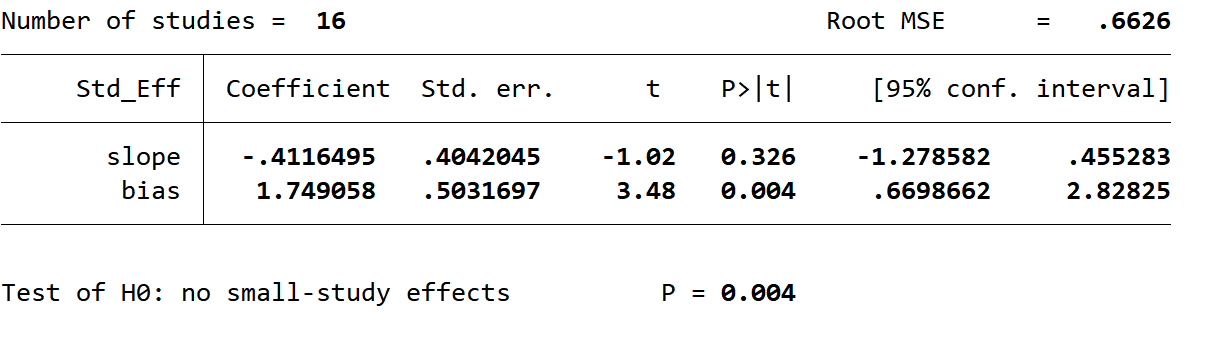

Supplement: Supplementary file 1 [file Data_Sheet_1.docx]
